# Melhora da Pressão Arterial após Jejum Intermitente na Hipertensão: O Sistema Renina-Angiotensina e o Sistema Nervoso Autônomo Podem Funcionar?

**DOI:** 10.36660/abc.20220756

**Published:** 2023-04-10

**Authors:** Erkan Demirci, Bekir Çalapkorur, Oguzhan Celik, Derya Koçer, Selami Demirelli, Ziya Şimsek

**Affiliations:** 1 Kayseri City Hospital Departamento de Cardiologia Kayseri Turquia Kayseri City Hospital – Departamento de Cardiologia, Kayseri – Turquia; 2 Mugla Sitki Kocman University Faculdade de Medicina Departamento de Cardiologia Mugla Turquia Mugla Sitki Kocman University, Faculdade de Medicina – Departamento de Cardiologia, Mugla – Turquia; 3 Kayseri City Hospital Departamento de Bioquímica Kayseri Turquia Kayseri City Hospital – Departamento de Bioquímica, Kayseri – Turquia

**Keywords:** Hipertensão, Jejum, Pressão Arterial, Sistema Renina Angiotensina, Sistema Nervoso Autônomo

## Abstract

**Fundamento:**

Embora tenha sido relatado que a dieta de jejum intermitente (JI) tem efeitos positivos na saúde do coração e na melhora da pressão arterial, ainda não foi suficientemente esclarecido como poderia ter esses efeitos positivos.Objetivo: Nosso objetivo foi avaliar os efeitos do JI no sistema nervoso autônomo (SNA) e no sistema renina-angiotensina (SRA), que estão intimamente relacionados à pressão arterial.

**Métodos:**

Setenta e dois pacientes hipertensos foram incluídos no estudo, e os dados de 58 pacientes foram usados. Todos os participantes jejuaram por cerca de 15-16 horas por 30 dias. Os participantes foram avaliados com monitorização ambulatorial da pressão arterial de 24 horas e eletrocardiograma Holter antes e após o JI; também, amostras de sangue venoso de 5 ml foram coletadas para avaliação dos níveis séricos de angiotensina I (Ang-I) e angiotensina II (Ang-II) e da atividade da enzima conversora de angiotensina (ECA). Para análise dos dados, o valor de p < 0,05 foi aceito como significativo.

**Resultados:**

Comparado ao pré-JI, observou-se queda significativa nas pressões arteriais dos pacientes no pós-JI. Um aumento na potência de alta frequência (AF) e na raiz quadrada média da soma dos quadrados das diferenças entre intervalos NN adjacentes (RMSSD) foram observados após o protocolo JI (p=0,039, p=0,043). A Ang-II e a atividade da ECA foram menores em pacientes após JI (p=0,034, p=0,004), e níveis decrescentes de Ang-II foram determinados como fatores preditivos para melhora da pressão arterial, como o aumento da potência de AF e RMSSD.

**Conclusão:**

Os presentes achados de nosso estudo demonstraram uma melhora na pressão arterial e a relação da pressão arterial com resultados positivos, incluindo VFC, atividade da ECA e níveis de Ang-II após o protocolo JI.

## Introdução

A hipertensão continua sendo a principal causa evitável de doença cardiovascular (DCV) e morte em todo o mundo.^1^ Os estudos sobre o mecanismo fisiopatológico e o tratamento ideal da hipertensão têm se concentrado principalmente no sistema renina-angiotensina (SRA) e no sistema nervoso autônomo (SNA). Embora não haja causa subjacente definitiva na hipertensão essencial (primária), há superativação do SRA e instabilidade autonômica (aumento da atividade simpática, diminuição da atividade parassimpática) na maioria dos casos. Existe uma interação complexa e bidirecional entre esses dois sistemas em condições fisiológicas e fisiopatológicas como a hipertensão. Além disso, os medicamentos anti-hipertensivos mais atuais visam suprimir a atividade excessiva desses dois sistemas.^2^

O SRA era originalmente conhecido como um sistema endócrino que regula a pressão sanguínea e o equilíbrio hidroeletrolítico.^3^ O SRA clássico é uma série de interações enzima-substrato nas quais a proteína substrato angiotensinogênio é processada em uma reação de duas etapas pela conversão de renina e angiotensina enzima (ECA) para produzir hormônios peptídicos funcionais chamados angiotensina I (Ang-I) e angiotensina II (Ang-II), respectivamente. A Ang-II é a molécula mais funcional do SRA e desempenha um papel ativo em muitos processos fisiológicos e patológicos. Aumenta a pressão arterial principalmente por vasoconstrição, inflamação, secreção de vasopressina e aldosterona, estresse oxidativo, proliferação celular e ativação imunológica por meio de receptores tipo I (AT1) da superfície celular.^4^ Além disso, a Ang-II contribui para a regulação da pressão arterial modulando o sistema nervoso autônomo nos níveis central e periférico.^5^ A ativação do braço do receptor ECA/Ang-II/AT1 do SRA causa deterioração na regulação autonômica cardiovascular ao aumentar a condução nervosa simpática, inibindo o tônus parassimpático-vagal cardiovascular e diminuindo a sensibilidade do barorreflexo.^6^

Sabe-se que a combinação de terapias farmacológicas e não farmacológicas é oferecida para o manejo da hipertensão, incluindo modificações no estilo de vida, como dietas de forma eficaz. O jejum intermitente (JI) é uma das dietas populares que demonstraram ter um efeito positivo na pressão arterial, e os protocolos JI são classificados como alimentação com restrição de tempo (JRT), jejum em dias alternados (JDA), dieta 5:2, e jejum do Ramadã (JR).^7 - 10^

Tem sido sugerido que o jejum intermitente melhora os fatores de risco cardiovascular por meio de três mecanismos possíveis: diminuição do estresse oxidativo, sincronização com o sistema circadiano e aumento da cetogênese.^11 - 13^ Além disso, uma diminuição na pressão arterial sistólica/diastólica foi relatada com o JI,^14^ mas ainda não há consenso sobre como o JI reduz a pressão arterial. Um dos possíveis mecanismos poderia ser uma diminuição do tônus simpático cardiovascular e um aumento do tônus parassimpático, o que corrige significativamente o desequilíbrio autonômico observado na maioria dos pacientes hipertensos.^15 , 16^ A modulação do SNA por JI também pode teoricamente causar supressão do vasoconstritor (ECA- Ang II-AT1 receptor) do SRA, dadas as interações do ANS e do SRA. Para apoiar esta hipótese, considerando as informações existentes na literatura, nosso estudo teve como objetivo avaliar 1. O efeito do JR na pressão arterial, 2. O efeito da JR no SRA medindo a atividade sérica da ECA e Ang-I e Ang- níveis II. 3. O efeito da JR no SNA medindo a variabilidade da frequência cardíaca (VFC) em pacientes hipertensos.

## Métodos

### Participantes

Em análises de poder, um bicaudal com tamanho de efeito de 0,5 pontos, um alfa de 0,05 e um poder de 0,80 precisou de um tamanho de amostra de apenas 34 para detectar esse efeito. Planejamos incluir cerca de 60 pacientes em nosso estudo, considerando o número de pacientes em um estudo realizado pela *Society of Hypertension and Renal Diseases* .^17^

Setenta e dois pacientes com idade entre 40 e 60 anos, que procuraram o ambulatório de cardiologia, apresentavam hipertensão controlada, usavam apenas bloqueadores dos canais de cálcio do grupo diidropiridínico como tratamento anti-hipertensivo, faziam qualquer outro tratamento medicamentoso por qualquer motivo e estavam em jejum voluntário. Os dados de 58 pacientes foram usados excluindo 14 pacientes que pararam de jejuar por mais de 2 dias por motivos diversos e não compareceram para controle ( Figura Central ).

**Figura Central:**
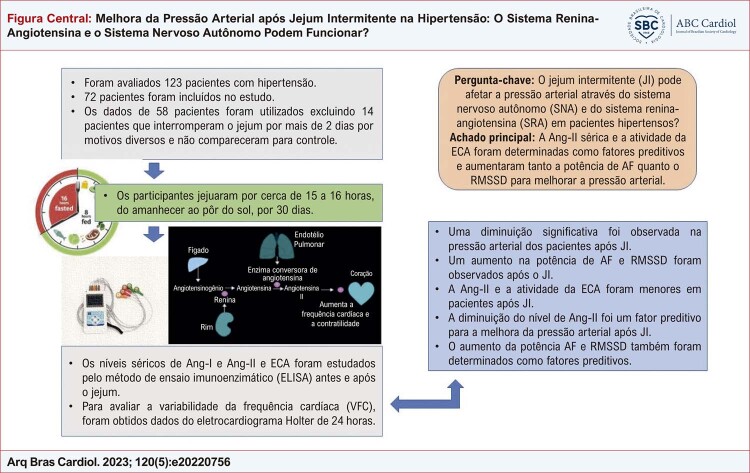
: Melhora da Pressão Arterial após Jejum Intermitente na Hipertensão: O Sistema Renina-Angiotensina e o Sistema Nervoso Autônomo Podem Funcionar?

Pacientes com pressão arterial desregulada, que apresentavam fatores de risco cardiovascular (tabagismo, IMC > 30 kg/m^2^ , hiperlipidemia, diabetes) que podem afetar a atividade do SRA e do SNA, aqueles com TFG < 50 e aumento duplo normal nos testes de função hepática, e que tinham doença cardiovascular, incluindo doença arterial coronariana, insuficiência cardíaca, insuficiência renal crônica e doença cerebrovascular foram excluídos do estudo. Pacientes em uso de drogas que podem afetar o SRA, a atividade do SNA (IECA, BRA, Diuréticos, β/α-bloqueadores) e PCR (estatina) também foram excluídos.

Em nosso estudo, utilizamos a JR como protocolo de JI. Os participantes jejuaram por cerca de 15 a 16 horas, do amanhecer ao pôr do sol, por 30 dias. Não houve nenhum paciente cujo tratamento foi alterado durante a JI. O motivo da escolha do JR é a facilidade de aplicação, não havendo restrição calórica (alimentação ad libitum) fora do período de jejum; portanto, não há necessidade de um cálculo estrito de calorias. Para limitar a variabilidade da ingestão de calorias, os participantes foram aconselhados a seguir uma rotina de 30 dias de comer a refeição principal após o pôr do sol e uma refeição leve antes do nascer do sol. Os participantes tomaram a medicação antes do início da inanição.

Os participantes foram verificados duas vezes, 5 dias antes do jejum e nos últimos 5 dias de jejum. Em seus controles, os participantes foram avaliados com monitoramento ambulatorial da pressão arterial de 24 horas e eletrocardiograma Holter. Em ambos os controles, amostras de 5 ml de sangue venoso foram coletadas entre 8:00 e 8:30 para testes bioquímicos.

O consentimento informado assinado foi obtido de todos os participantes antes de participar do estudo. Este estudo foi aprovado pelo comitê de ética local do Hospital Municipal (2021/517).

### Análises bioquímicas de amostras de sangue

Amostras de sangue foram coletadas dos pacientes sentados após 20 minutos de repouso após 12 horas de jejum. A veia antecubital foi utilizada para obtenção da amostra de sangue. O soro e o plasma foram separados após as amostras serem centrifugadas por 10 minutos a 5000 rpm (centrífugas NF 400, Turquia). Eles foram mantidos a -80 °C até que os ensaios dos parâmetros fossem realizados por um bioquímico clínico experiente.

Os níveis séricos de angiotensina 1 e angiotensina 2 e a atividade da ECA foram estudados pelo método de ensaio imunoenzimático (ELISA) usando kits comerciais (Bioassay Technology Laboratory, Xangai, China). Os níveis de Ang-I e Ang-II e a atividade da ECA foram analisados de acordo com as instruções do fabricante e expressos em ng/L, ng/L e U/L, respectivamente.

### Avaliação da variabilidade da pressão arterial e da frequência cardíaca

Para avaliar a pressão arterial dos participantes, foram realizadas duas medidas de pressão arterial ambulatorial de 24 horas, 5 dias antes da JI e nos últimos 5 dias da JI. Foram obtidos os níveis médios de pressão arterial sistólica e diastólica de 24 horas e os níveis médios diurnos/noturnos de pressão arterial sistólica e diastólica. Para avaliar a variabilidade da frequência cardíaca (VFC), os dados do eletrocardiograma Holter de 24 horas foram obtidos duas vezes (modelo Ge: informações do software SEER 100 MARS). Os parâmetros e medidas da VFC foram feitos com base nos pareceres da European Society of Cardiology e da North American Society of Battery and Electrophysiology. O desvio padrão de todos os intervalos RR [NN] normais a normais (SDNN), a raiz quadrada média da soma dos quadrados das diferenças entre intervalos NN adjacentes (RMSSD), e o número de pares de intervalos NN que diferem em mais de 50 ms (pNN50) em 24 horas, potência de baixa frequência (BF), potência de alta frequência (AF) e a proporção dos dois (BF/AF) foram obtidos a partir de registros de ECG de 24 horas. Além disso, foram obtidas a frequência cardíaca máxima, a frequência cardíaca mínima, a frequência cardíaca média de 24 horas e a frequência cardíaca noturna média dos pacientes.

### Análise estatística

Os dados foram analisados usando o IBM SPSS Statistics 21.0. A distribuição dos dados foi analisada por meio do teste Shapiro-Wilk e QQ Plot Normality. Frequências e porcentagens foram calculadas para variáveis categóricas. Variáveis contínuas com distribuição normal foram expressas como média e desvio padrão, enquanto variáveis contínuas com distribuição não normal foram expressas como mediana e intervalo interquartílico. Para comparar as variáveis, foram utilizados o teste t de amostras pareadas e o teste de Wilcoxon. Os efeitos preditivos dos parâmetros sobre a pressão arterial foram avaliados pelo método de análise de regressão linear. A adequação da regressão linear ao modelo de 6 etapas foi controlada em modelos para PAS e PAD (para PAS; Durbin-Watson; 1,981, Std. Residual; -1,879-1,934, distância de Cook; 0,000-0,375, para DBP, Durbin-Watson; 1.653, Std. Residual; -1.537-2.178, Distância de Cook; 0,000-0,433). Altos níveis de correlação excluíram BUN, ácido úrico e níveis de colesterol total das análises. Um valor de p < 0,05 foi aceito como significativo nas análises estatísticas.

## Resultados

Cinquenta e oito pacientes com HTA controlada entre as idades de 40-60 foram incluídos no estudo. As características dos pacientes são dadas na Tabela 1 .

**Tabela 1:** – Características dos pacientes

	Pacientes com HTA (N:58)
**Sexo, Masculino, n (%)**	35 (60,34)
**Idade (Anos)**	49,3 ± 8,7
**Duração da HTA (anos)**	4,7±2,6

A Ang-I sérica foi menor, enquanto os níveis de Ang-II e ECA foram maiores nos hipertensos pré-JI do que nos hipertensos pós-JI ( Tabela 2 ). Em pacientes hipertensos antes e depois da JI, não houve alteração estatisticamente significativa no IMC, glicemia de jejum, HgA1C, creatinina, TFG, BUN, ácido úrico, colesterol total, LDL, HDL, TG e níveis de TSH (p>0,05 para todos), enquanto uma diminuição significativa foi observada nos níveis de PCR após a JI ( Tabela 3 ). Em relação ao pré-JI, observou-se diminuição significativa das médias das pressões arteriais sistólica e diastólica aferidas às 24 horas e à noite nos hipertensos pós-JI ( Tabela 4 ). Observou-se diminuição nos valores da Frequência Cardíaca Máxima e da Frequência Cardíaca Média (24 Horas) após a JI, embora os valores da Frequência Cardíaca Mínima e da Frequência Cardíaca Média Noturna não tenham mudado estatisticamente ( Tabela 4 ). A potência de IC, o valor de LF/HF e os níveis de RMSSD diferem significativamente em pacientes hipertensos pós-IF em comparação com pré-IF ( Tabela 4 ).

**Tabela 2:** – Comparação de biomarcadores antes e após JI em pacientes com HTA

	Antes de JI 25-75 50º (mediana)	Depois JI 25-75 50º (mediana)	Comparação
**ECA(U/L)**	38.02-51.98 44,97	34,61-44,42 39.38	**p=0,004**
**Ang-I (ng/L)**	64,93-135,61 96,46	89,18-146,30 103,74	**p=0,043**
**Ang-II (ng/L)**	37.15-46.57 43,70	32,84-44,22 40,78	**p=0,034**

*ECA: enzima conversora de angiotensina I; Ang-I: angiotensina I; ANG-II: angiotensina II. ***Teste de Wilcoxon****

**Tabela 3:** – Comparação das características clínicas antes e depois do JI em pacientes com HTA

	Antes de JI *Média±DP*	Depois JI *Média±DP*	Comparação
**IMC (kg/m** ^**2**^ **)**	26,05±3,03	26,59±3,05	p=0,425
**Glicemia em jejum (mg/dL)**	109,06±33,95	108,86±35,98	p=0,992
**HgA1C (%)**	5,95±0,62	5,88±0,69	p=0,492
**Creatinina (mg/dL)**	0,79 ± 0,17	0,80 ± 0,15	p=0, 143
**TFG, (ml/min/1,73m** ^**2**^ **)**	86,3 ± 16,2	83,4 ± 15,5	p=0, 124
**BUN, (mg/dL)**	15,68 ± 5,36	16,68 ± 5,43	p=0,232
**Ácido úrico (mg/dL)**	5,74± 1,22	6,19±1,26	p=0,092
**Colesterol Total, (mmol/L)**	206,77± 34,12	196,31 ± 29,82	p=0,087
**LDL (mg/dL)**	137,81 ± 29,10	128,86 ± 27,76	p=0,129
**HDL (mg/dL)**	46,59 ± 11,37	44,22 ± 10,83	p=0,110
**Triglicéride, (mg/dL)**	177,2 ± 51,32	182,3 ± 48,23	p=0,095
**PCR (mg/L)**	4,58±1,93	3,44±1,29	**p=0,016**
**TSH (mIU/L)**	3,06±0,31	3,08±0,38	p=0,475

*IMC: índice de massa corporal; LDL: lipoproteína de baixa densidade; HDL: lipoproteína de alta densidade; PCR: proteína C reativa. ***Teste t pareado****

**Tabela 4:** – Comparação da variabilidade da pressão arterial e da frequência cardíaca antes e depois da JI em pacientes com HTA

	Antes de JI *Média±DP*	Depois JI *Média±DP*	Comparação
**Pressão arterial sistólica (24 horas)**	139,48±12,26	126,44±7,93	**p<0,001**
**Pressão arterial diastólica (24 horas)**	84,26±7,54	76,35±5,36	**p=0,014**
**Pressão arterial sistólica noturna**	133,35±15,46	123,45±10,56	**p<0,001**
**Pressão sanguínea diastólica noturna**	79,45±10,64	71,35±6,45	**p<0,001**
**Frequência cardíaca máxima (batidas/min)**	156,21±13,55	148,42±14,60	**p=0,041**
**Frequência cardíaca mínima (batidas/min)**	65,43±10,42	64,86±10,25	p=0,634
**Frequência cardíaca média (24 horas)**	86,93 ± 10,44	74,55 ± 12,78	**p=0,042**
**Frequência cardíaca média Noturna**	69,48±10,51	72,70±10,26	p=0,067
**Potência de alta frequência**	193,93±63,02	216,00±76,07	**p=0,039**
**Valor BF/AF**	4,75±1,36	3,95±1,49	**p=0,041**
**SDNN (ms)**	126,16 ± 32,85	137,48 ± 30,56	p=0,059
**RMSSD (ms)**	33,76 ± 14,39	37,02 ± 11,35	**p=0,042**
**pNN50 (%)**	6,68 ±2,51	8,20±2,91	p=0,063

*Potência de alta frequência (AF), relação de frequência baixa-alta (BF/AF), SDNN (desvio padrão de todos os intervalos RR [NN] normais a normais), RMSSD (a raiz quadrada média da soma dos quadrados das diferenças entre intervalos NN adjacentes), pNN50 (o número de pares de intervalos NN que diferem em mais de 50 ms). ***Teste t pareado****

Considerando os efeitos do jejum intermitente no perfil lipídico IMC e PCR na literatura, as relações do IMC e TSH com os níveis de ECA e a hipótese do estudo, todos os dados foram (exceto para dados de pressão arterial, BUN, ácido úrico e níveis de colesterol total) incluídos na análise de regressão para diminuir a pressão arterial sistólica e diastólica. O modelo de regressão foi estatisticamente significativo (p=0,032), e a diminuição da atividade da ECA e dos níveis de Ang-II, aumentando também o RMSSD e a potência de IC, foram avaliados como fatores preditivos para a diminuição da pressão arterial sistólica após a JI ( Tabela 5 ). Além disso, no mesmo modelo utilizado para a pressão arterial diastólica (p = 0,46), apenas a diminuição do nível de Ang-II foi encontrada como fator preditivo para a diminuição da pressão arterial diastólica após a JI (p = 0,031).

**Tabela 5:** – Preditores potenciais de mudanças na pressão arterial sistólica no modelo de regressão

	B	Intervalo de confiança de 95% para B		valor-p

		Inferior	Superior	
**ECA**	-0,113	-0,787	0,144	**0,044**
**Ang-II**	-0,318	-0,606	-0,029	**0,032**
**PCR**	-0,320	-1,481	0,840	0,081
**Frequência cardíaca máxima (batidas/min)**	-0,242	-0,525	0,042	0,063
**Potência de alta frequência**	0,293	0,837	1,023	**0,041**
**Valor BF/AF**	-0,017	-0,119	0,409	0,077
**RMSSD (ms)**	0,048	-0,902	0,951	**0,037**

*Método=Retroceder. R^*2*^ ajustado; %61,2, Durbin-Watson; 1.981, Std. Residual; -1.879-1.934, Distância de Cook;.000-.375*

## Discussão

Pesquisas em humanos indicam que o JI pode ter vantagens cardiovasculares. O JI parece afetar positivamente inúmeras variáveis de risco cardiovascular, incluindo HTA, enquanto os mecanismos subjacentes são desconhecidos. Nosso estudo é um dos poucos que avaliaram os possíveis mecanismos subjacentes ao efeito da JR, um dos subtipos de JI, sobre a pressão arterial. É também o primeiro estudo em que os sistemas SRA e ANS, reconhecidamente importantes na regulação da pressão arterial, foram avaliados em conjunto. Os presentes achados de nosso estudo demonstraram os efeitos do JI na pressão arterial e na VFC. Outro fator principal que apresentou evolução positiva após o protocolo de JI foi a atividade da ECA e os níveis de Ang-II.

Estudos demonstraram que a terapia combinada com JR afeta positivamente a pressão arterial diurna em pacientes hipertensos.^18^ Por outro lado, resultados conflitantes foram relatados em estudos que não encontraram nenhuma diferença.^19^ Além disso, benefícios cardiovasculares e metabólicos, como diminuição de TG, LDL, massa gorda e PCR, foram relatados no jejum terapêutico.^20^ Além disso, melhorias nos indicadores de saúde cardiovascular podem ser observadas 2 a 4 semanas após o início do JI em estudos animais.^15^ Em nosso estudo, em comparação com o pré-JI, foi observada uma diminuição significativa nas pressões arteriais médias sistólica e diastólica medidas em 24 horas e à noite em pacientes hipertensos pós-JI. Além das diferenças metodológicas entre os estudos, a inclusão de hipertensos controlados em nosso estudo pode ter levado a uma melhora mais significativa dos níveis pressóricos, uma vez que os casos resistentes foram excluídos. Não houve alteração estatisticamente significativa na glicemia de jejum, HgA1C, creatinina, GFR, BUN, colesterol total, LDL, HDL e níveis de TG, enquanto uma diminuição significativa foi observada nos níveis de PCR após a JI. No entanto, os níveis de PCR não foram avaliados como fator preditivo para melhorar os valores da pressão arterial nas análises de regressão. Além disso, não observamos nenhuma mudança estatisticamente significativa no IMC. Em um estudo de metanálise, foi relatado que o jejum intermitente teve efeitos positivos no IMC e na proporção de gordura.^21^ Por outro lado, considerando os estudos que os tempos de dieta são em sua maioria superiores a 1 mês, a razão pela qual não houve mudança significativa no IMC valores em nosso estudo podem ser a duração. É evidente a necessidade de novos estudos avaliando os efeitos do JI sobre o IMC em longo prazo.

Pacientes com hipertensão essencial, especialmente aqueles não medicados, têm um aumento na atividade simpática e uma diminuição na atividade parassimpática do sistema nervoso autônomo.^22^ O RMSSD é a métrica chave no domínio do tempo usada para avaliar as alterações vagalmente mediadas observadas na VFC. Reflete a variação batimento a batimento na FC.^23^ As leituras de RMSSD ao longo de 24 horas estão altamente associadas com pNN50 e potência de AF.^24^ Estudos limitados investigaram os efeitos do JI na VFC em pacientes com HTA.^25 , 26^ Em um estudo, VFC foi avaliado duas vezes por registros ambulatoriais de Holter de 24 horas em jejum durante e após JI em 20 pacientes hipertensos com ritmo sinusal que variavam em fatores de estilo de vida. Considerando as variações estatisticamente significativas em SDNN, SDANN, BF e potência T entre os dois grupos, foi sugerido que a JR reduz a atividade do sistema nervoso simpático.^25^ Por outro lado, em outro estudo que incluiu 58 pacientes hipertensos, a JR aumentou significativamente a VFC e reduziu o estresse cardíaco entre os pacientes controlados por um aderente à medicação hipertensiva.^25^ Em nosso estudo, enquanto uma diminuição foi observada na Frequência Cardíaca Máxima após a JI, os valores da Frequência Cardíaca Mínima e da Frequência Cardíaca Média Noturna diminuíram, mas não se alteraram estatisticamente. Comparado ao pré-JI, um aumento na potência de IC e nos níveis de RMSSD e uma diminuição em BF/AF foram observados em pacientes hipertensos pós-JI. Como resultado importante, os aumentos de RMSSD e de potência de AF foram avaliados como fatores preditivos para a diminuição da pressão arterial sistólica após a JI.

A Ang-I é convertida em Ang-II pela ECA zinco metalopeptidase.^27^ O efeito da Ang-II no aumento da pressão arterial e na retenção de sal e água é bem conhecido.^28^ Embora estudos em animais tenham relatado que o JI pode ter efeitos positivos no SRA em Na literatura, nenhum estudo avaliou os efeitos do JI sobre o SRA em pacientes hipertensos. Em um estudo com animais, Camelo et al.,^29^ levantaram a hipótese de que o JI reduz a pressão arterial e melhora o perfil lipídico em camundongos devido a um SRA local menos ativado no ventrículo esquerdo, independentemente do plano alimentar.^29^ Eles descobriram que a perda de peso causada pelo tratamento com JI resultou na regulação do SRA local, com o benefício do remodelamento do ventrículo esquerdo (VE) e redução da pressão arterial.^29^ Em outro estudo com ratos, foram investigados os efeitos dos esquemas de JI sobre o nível plasmático de Ang-II, a expressão dos receptores Ang-II e ECA2.^30^ Os animais idosos demonstraram ter maior índice de hipertrofia cardíaca. O coração e a aorta apresentaram maior expressão de AT1aR e menor expressão de AT2R. Aumentando os parâmetros declarados e o equilíbrio do SRA, “jejuar a cada dois dias” foi mais eficaz do que “jejuar um dia por semana”.^30^ Em nosso estudo, a Ang-I sérica foi maior, enquanto a Ang-II e a atividade da ECA foram menores nos pacientes hipertensos pós-JI do que nos pacientes hipertensos pré-JI, e a diminuição da atividade da ECA e os níveis de Ang-II foram determinados como fatores preditivos para a diminuição da pressão arterial sistólica após a JI. Além disso, a diminuição do nível de Ang-II foi um fator preditivo para a diminuição da pressão arterial diastólica após a JI. Outro ponto a ser enfatizado é que a atividade da ECA está associada ao IMC e às funções tireoidianas.^31 , 32^ Portanto, pacientes eutireoideos foram incluídos em nosso estudo e não houve alteração estatisticamente significativa no IMC e nos níveis de TSH antes e após a JI. Para controlar essas variáveis, os níveis de IMC e TSH foram adicionados à análise de regressão no modelo criado para a mudança da pressão arterial, e nenhuma relação significativa foi encontrada.

Existem algumas limitações do nosso estudo. O número de participantes foi pequeno, pois apenas pacientes em uso de bloqueadores dos canais de cálcio do grupo diidropiridínico foram incluídos no estudo como tratamento anti-hipertensivo. Os participantes consistiam apenas em pacientes com hipertensão controlada e baixo risco cardiovascular. Futuros estudos abrangentes são necessários para determinar se as aplicações de FI são eficazes em pacientes com maior risco cardiovascular (como diabetes, doença arterial coronariana, insuficiência renal crônica e doença cerebrovascular) com maior atividade de SRA e SNA.

## Conclusões

Em conclusão, os resultados do nosso estudo podem ser interpretados como: 1. A ativação do sistema parassimpático avaliada pela VFC desempenha um papel nos efeitos positivos do JI sobre a pressão arterial. 2. A diminuição dos níveis de Ang-II pode ser aceita como uma das razões para melhorar a hipertensão sobre a regulação da ativação do sistema simpático sobre o SRA.
