# Supplementary material for: Melhora da Pressão Arterial após Jejum Intermitente na Hipertensão: O Sistema Renina-Angiotensina e o Sistema Nervoso Autônomo Podem Funcionar?
Source: Arq Bras Cardiol. 2023 Apr 10;120(5):e20220756. [Article in Portuguese] doi: 10.36660/abc.20220756 (PMC10124600; doi:10.36660/abc.20220756)
Supplement: Translation

# Translation: Improvement in Blood Pressure After Intermittent Fasting in Hipertension: Could Renin-Angiotensin System and Autonomic Nervous System Have a Role?

**Authors:** Erkan Demirci, Bekir Çalapkorur, Oguzhan Celik, Derya Koçer, Selami Demirelli, Ziya Şimsek

**Affiliations:** 1 Turquia Kayseri City Hospital – Departamento de Cardiologia, Kayseri – Turquia; 2 Turquia Mugla Sitki Kocman University, Faculdade de Medicina – Departamento de Cardiologia, Mugla – Turquia; 3 Turquia Kayseri City Hospital – Departamento de Bioquímica, Kayseri – Turquia

**Keywords:** Hypertension, Fasting, Blood Pressure, Renin-Angiotensin System, Autonomic Nervous System

## Abstract

**Background:**

Although it has been reported that the intermittent fasting (IF) diet has positive effects on heart health and improvement in blood pressure, it has not been sufficiently clarified how it could have these positive effects yet.

**Objective:**

We aimed to evaluate the effects of IF on the autonomic nervous system (ANS) and renin-angiotensin system (RAS), which are closely related to blood pressure.

**Methods:**

Seventy-two hypertensive patients were included in the study, and the data of 58 patients were used. All the participants fasted for about 15-16 hours for 30 days. Participants were evaluated with 24-hour ambulatory blood pressure monitoring and Holter electrocardiography before and after IF; also, 5 ml venous blood samples were taken for assessment of Serum angiotensin I (Ang-I) and angiotensin II (Ang-II) levels and angiotensin-converting enzyme (ACE) activity. For data analysis, the p-value <0.05 was accepted as significant.

**Results:**

Compared to pre-IF, a significant decrease was observed in the patients’ blood pressures in post-IF. An increase in high-frequency (HF) power and the mean root square of the sum of squares of differences between adjacent NN intervals (RMSSD) were observed after the IF protocol (p=0.039, p=0.043). Ang-II and ACE activity were lower in patients after IF (p=0.034, p=0.004), and decreasing Ang-II levels were determined as predictive factors for improvement of the blood pressure, like the increase in HF power and RMSSD.

**Conclusion:**

The present findings of our study demonstrated an improvement in blood pressure and the relationship of blood pressure with positive outcomes, including HRV, ACE activity, and Ang-II levels after the IF protocol.

## Introduction

Hypertension remains the leading preventable cause of cardiovascular disease (CVD) and death worldwide.^1^ Studies on the pathophysiological mechanism and optimal treatment of hypertension have focused mainly on the renin-angiotensin system (RAS) and the autonomic nervous system (ANS). Although there is no definitive underlying cause in essential (primary) hypertension, there is overactivation of the RAS and autonomic instability (increased sympathetic activity, decreased parasympathetic activity) in most cases. There is a complex and bidirectional interaction between these two systems in physiological and pathophysiological conditions such as hypertension. Also, most current antihypertensive drugs aim to suppress the excessive activity of these two systems.^2^

The RAS was originally known as an endocrine system that regulates blood pressure and fluid-electrolyte balance.^3^ Classical RAS is a series of enzyme-substrate interactions in which the substrate protein angiotensinogen is processed in a two-step reaction by renin and angiotensin-converting enzyme (ACE) to produce functional peptide hormones called angiotensin I (Ang-I) and angiotensin II (Ang-II), respectively. Ang-II is the most functional molecule of RAS and plays an active role in many physiological and pathological processes. It raises blood pressure mainly by vasoconstriction, inflammation, vasopressin and aldosterone secretion, oxidative stress, cellular proliferation, and immunological activation through cell surface type I (AT1) receptors.^4^ Also, Ang-II contributes to regulating blood pressure by modulating the autonomic nervous system at both central and peripheral levels.^5^ Activation of the ACE/Ang-II/AT1 receptor arm of the RAS causes deterioration in cardiovascular autonomic regulation by increasing sympathetic nerve conduction, inhibiting cardiovascular parasympathetic-vagal tone, and decreasing baroreflex sensitivity.^6^

It is known that combining pharmacological and non-pharmacological therapies is offered to manage hypertension, including lifestyle modifications such as diets effectively. Intermittent fasting (IF) is one of the popular diets that has been shown to have a positive effect on blood pressure, and IF protocols are classified as time-restricted feeding (TRF), alternate-day fasting (ADF), 5:2 diet, and Ramadan fasting (RF).^7 - 10^

It has been suggested that IF improves cardiovascular risk factors through three possible mechanisms: decreasing oxidative stress, synchronization with the circadian system, and increasing ketogenesis.^11 - 13^ Also, a decrease in systolic/diastolic blood pressure was reported with IF,^14^ but there is no consensus yet on how IF reduces blood pressure. One of the possible mechanisms could be a decrease in cardiovascular sympathetic tone and an increase in parasympathetic tone, which significantly corrects the autonomic imbalance observed in most hypertensive patients.^15 , 16^ ANS modulation by IF may also theoretically cause suppression of the vasoconstrictor (ACE-Ang II-AT1 receptor) arm of the RAS, given the interactions of the ANS and the RAS. In order to support this hypothesis, considering the information in the existing literature, our study aimed to evaluate 1. The effect of RF on blood pressure, 2. The effect of RF on RAS by measuring serum ACE activity and Ang-I and Ang-II levels. 3. The effect of RF on ANS by measuring heart rate variability (HRV) in hypertensive patients.

## Methods

### Participants

In power analyses, a two-tailed with 0.5 point effect size, an alpha of .05, and a power of .80 needed a sample size of only 34 to detect that effect. We planned to include about 60 patients in our study, considering the number of patients in a study conducted by the Society of Hypertension and Renal Diseases.^17^

Seventy-two patients aged 40-60 years, who applied to the cardiology outpatient clinic, had controlled hypertension, used only dihydropyridine group calcium channel blockers as antihypertensive treatment, had any other drug treatment for any reason, and voluntarily fasted were included in our study. The data of 58 patients were used by excluding 14 patients who stopped fasting for more than 2 days for various reasons and did not come for control ( Central Illustration ).

**Central Illustration:** : Improvement in Blood Pressure After Intermittent Fasting in Hipertension: Could Renin-Angiotensin System and Autonomic Nervous System Have a Role?

Patients with unregulated blood pressure, who had cardiovascular risk factors (smoking, BMI> 30 kg/m2, hyperlipidemia, diabetes) that may affect RAS and ANS activity, those with a GFR <50 and a double-normal increase in liver function tests, and who had cardiovascular disease including coronary artery disease, heart failure, chronic kidney failure, and cerebrovascular disease were excluded from the study. Patients using drugs that may affect RAS, ANS activity (ACEI, ARB, Diuretics, β/α-blockers), and CRP (statin) were also excluded.

In our study, we used RF as the IF protocol. Participants fasted for about 15-16 hours from dawn to sunset for 30 days. There was no patient whose treatment was changed during IF. The reason for choosing the RF is that it is easy to apply, and there is no calorie restriction (ad libitum nutrition) outside the fasting period; therefore, there is no need for a strict calorie calculation. To limit calorie intake variability, participants were advised to follow a 30-day routine of eating the main meal after sunset and a light meal before sunrise. Participants took their medication before the onset of starvation.

Participants were checked twice, 5 days before fasting and in the last 5 days of fasting. In their controls, participants were evaluated with 24-hour ambulatory blood pressure monitoring and Holter electrocardiography. In both controls, 5 ml venous blood samples were taken between 08:00 and 08:30 for biochemical tests.

Signed informed consent was obtained from all participants before participating in the study. This study was approved by the local ethics committee of the City Hospital (2021/517).

### Biochemical analyzes of blood samples

Blood samples were taken from the patients seated after a 20 min rest following 12h of fasting. The ante-cubital vein was used to obtain the blood sample. The serum and plasma were separated after the samples were centrifuged for 10 minutes at 5000 rpm (NF 400 centrifuges, Turkey). They were kept at −80 °C until assays for parameters were performed by an experienced clinical biochemist.

Serum angiotensin 1 and angiotensin 2 levels and ACE activity were studied by enzyme-linked immunosorbent assay (ELISA) method using commercial kits (Bioassay Technology Laboratory, Shanghai, China). Ang-I and Ang-II levels and ACE activity were analyzed according to the manufacturer’s instructions and expressed as ng/L, ng/L, and U/L, respectively.

### Assessment of blood pressure and heart rate variability

In order to evaluate the blood pressure of the participants, two 24-hour ambulatory blood pressure measurements were made 5 days before IF and in the last 5 days of IF. 24-hour mean systolic and diastolic blood pressure levels and day/night mean systolic and diastolic blood pressure levels were obtained. To assess heart rate variability (HRV), 24-hour Holter electrocardiography data were obtained twice (Ge model: SEER 100 software information MARS). HRV parameters and measurements were made based on the opinions of the European Society of Cardiology and the North American Society of Battery and Electrophysiology. The standard deviation of all normal to normal RR [NN] intervals (SDNN), the root mean square of the sum of squares of differences between adjacent NN intervals (RMSSD), and the number of pairs of NN intervals that differ by more than 50 ms (pNN50) in 24 hours, low-frequency (LF) power, high-frequency (HF) power, and the ratio of the two (LF/HF) were obtained from 24-hour ECG recordings. In addition, the patients’ maximal heart rate, minimal heart rate, 24-hour mean heart rate, and mean nightly heart rate were obtained.

### Statistical analysis

The data were analyzed using IBM SPSS Statistics 21.0. The data distribution was analyzed using the Shapiro-Wilk test and Q-Q Plot Normality. Frequencies and percentages were calculated for categorical variables. Continuous variables with normal distribution were expressed as the mean and standard deviation, while continuous variables with non-normal distribution were expressed as the median and interquartile range. To compare variables, the paired-sample T-test and the Wilcoxon test were used. The predictive effects of parameters on blood pressure were evaluated using the linear regression analysis method. The suitability of the linear regression to the 6-step model was controlled in models for SBP and DBP (For SBP; Durbin-Watson; 1.981, Std. Residual; -1.879-1.934, Cook’s Distance; .000-.375, for DBP, Durbin-Watson; 1.653, Std. Residual; -1.537-2.178, Cook’s Distance; .000-.433). High correlation levels excluded BUN, Uric acid, and total cholesterol levels from analyses. A p-value <0.05 was accepted as significant in statistical analyses.

## Results

Fifty-eight patients with controlled HT between the ages of 40-60 were included in the study. The characteristics of the patients are given in Table 1 .

**Table 1:** – Characteristics of patients

	HT patients (N:58)
**Sex, Male, n (%)**	35 (60.34)
**Age (years)**	49.3 ± 8.7
**Duration of HT (years)**	4.7±2.6

Serum Ang-I was lower, while Ang-II and ACE levels were higher in the pre-IF hypertensive patients than in post-IF hypertensive patients ( Table 2 ). In hypertensive patients before and after IF, there was no statistically significant change in BMI, fasting blood glucose, HgA1C, creatinine, GFR, BUN, uric acid, total cholesterol, LDL, HDL, TG, and TSH levels (p>0.05 for all), while a significant decrease was observed in CRP levels after IF ( Table 3 ). Compared to pre-IF, a significant decrease was observed in the mean systolic and diastolic blood pressures measured at 24 hours and night in post-IF hypertensive patients ( Table 4 ). A decrease was observed in Maximum Heart Rate and Average Heart Rate (24 Hours) values after IF, although Minimum Heart Rate and Average Night Heart Rate values did not change statistically ( Table 4 ). HF power, LF/HF value, and RMSSD levels differ significantly in post-IF hypertensive patients compared to pre-IF ( Table 4 ).

**Table 2:** – Comparison of biomarkers before and after IF in patients with HT

	Before IF 25th-75th 50th (Median)	After IF 25th-75th 50th (Median)	Comparison
**ACE (U/L)**	38.02-51.98 44.97	34.61-44.42 39.38	**p=0.004**
**Ang-I (ng/L)**	64.93-135.61 96.46	89.18-146.30 103.74	**p=0.043**
**Ang-II (ng/L)**	37.15-46.57 43,70	32.84-44.22 40,78	**p=0.034**

*ACE: angiotensin-I-converting enzyme; Ang-I: angiotensin I; Ang-II: angiotensin II. ***Wilcoxon test****

**Table 3:** – Comparison of clinical characteristics before and after IF in patients with HT

	Before IF *Mean±SD*	After IF *Mean±SD*	Comparison
**BMI (kg/m** ^**2**^ **)**	26.05±3.03	26.59±3.05	p=0.425
**Fasting blood glucose (mg/dL)**	109.06±33.95	108.86±35.98	p=0.992
**HgA1C (%)**	5.95±0.62	5.88±0.69	p=0.492
**Creatinin (mg/dL)**	0.79 ± 0.17	0.80 ± 0.15	p=0. 143
**GFR, (ml/min/1.73m** ^**2**^ **)**	86.3 ± 16.2	83.4 ± 15.5	p=0. 124
**BUN, (mg/dL)**	15.68 ± 5.36	16.68 ± 5.43	p=0.232
**Uric acid (mg/dL)**	5.74± 1.22	6.19±1.26	p=0.092
**Total Cholesterol, (mmol/L)**	206.77± 34.12	196.31 ± 29.82	p=0.087
**LDL (mg/dL)**	137.81 ± 29.10	128.86 ± 27.76	p=0.129
**HDL (mg/dL)**	46.59 ± 11.37	44.22 ± 10.83	p=0.110
**Trigliserid, (mg/dL)**	177.2 ± 51.32	182.3 ± 48.23	p=0.095
**CRP (mg/L)**	4.58±1.93	3.44±1.29	**p=0.016**
**TSH (mIU/L)**	3.06±0.31	3.08±0.38	p=0.475

*BMI: body mass ındex, LDL: low-density lipoprotein, HDL: high-density lipoprotein, CRP: C-reactive protein. ***Paired t-test****

**Table 4:** – Comparison of blood pressure and heart rate variability before and after IF in patients with HT

	Before IF *Mean±SD*	After IF *Mean±SD*	Comparison
**Systolic blood pressure (24 Hours)**	139.48±12.26	126.44±7.93	**p<0.001**
**Diastolic blood pressure (24 Hours)**	84.26±7.54	76.35±5.36	**p=0.014**
**Night Systolic blood pressure**	133.35±15.46	123.45±10.56	**p<0.001**
**Night Diastolic blood pressure**	79.45±10.64	71.35±6.45	**p<0.001**
**Maximum Heart Rate (beats/min)**	156.21±13.55	148.42±14.60	**p=0.041**
**Minimum Heart Rate (beats/min)**	65.43±10.42	64.86±10.25	p=0.634
**Average Heart rate (24 Hours)**	86.93 ± 10.44	74.55 ± 12.78	**p=0.042**
**Average Heart Rate Night**	69.48±10.51	72.70±10.26	p=0.067
**HF power**	193.93±63.02	216.00±76.07	**p=0.039**
**LF/HF value**	4.75±1.36	3.95±1.49	**p=0.041**
**SDNN (ms)**	126.16 ± 32.85	137.48 ± 30.56	p=0.059
**RMSSD (ms)**	33.76 ± 14.39	37.02 ± 11.35	**p=0.042**
**pNN50 (%)**	6.68 ±2.51	8.20±2.91	p=0.063

*High-frequency (HF) power, the ratio of low-high frequency (LF/HF), SDNN (standard deviation of all normal to normal RR [NN] intervals), RMSSD (the mean root square of the sum of squares of differences between adjacent NN intervals), pNN50 (the number of pairs of NN intervals that differ by more than 50 ms). ***Paired t-test****

Considering the effects of intermittent fasting on lipid profile BMI and CRP in the literature, the relations of BMI and TSH with ACE levels, and the hypothesis of the study, all data were (except for blood pressure data, BUN, Uric acid and total cholesterol levels) included in the regression analysis for decreasing systolic and diastolic blood pressure. The regression model was statistically significant (p=0.032), and decreasing ACE activity and Ang-II levels, also increasing RMSSD and HF power, were assessed as predictive factors for decreasing systolic blood pressure after IF ( Table 5 ). In addition, in the same model used for diastolic blood pressure (p =0.46), only decreasing Ang-II level was found as a predictive factor for decreasing diastolic blood pressure after IF (p=0.031).

**Table 5:** – Potential predictors of changes in systolic blood pressure in the regression model

	B	95% confidence interval for B		p-value

		Lower	Upper	
**ACE**	-0.113	-0.787	0.144	**0.044**
**Ang-II**	-0.318	-0.606	-0.029	**0.032**
**CRP**	-0.320	-1.481	0.840	0.081
**Maximum Heart Rate (beats/min))**	-0.242	-0.525	0.042	0.063
**HF power**	0.293	0.837	1.023	0.041
**LF/HF value**	-0.017	-0.119	0.409	0.077
**RMSSD (ms)**	0.048	-0.902	0.951	0.037

*Method=Backward. Adjusted R^*2*^ ;%61.2, Durbin-Watson; 1.981, Std. Residual; -1.879-1.934, Cook’s Distance;.000-.375*

## Discussion

Human research indicates that IF may have cardiovascular advantages. IF appears to positively affect numerous cardiovascular risk variables, including HT, while the underlying mechanisms are unknown. Our study is one of the few studies that evaluated the possible mechanisms underlying the effect of RF, one of the subtypes of IF, on blood pressure. It is also the first study in which the RAS and ANS systems, which are known to be important in blood pressure regulation, were evaluated together. The present findings of our study demonstrated the effects of IF on blood pressure and HRV. Another main factor that presented a positive outcome after the IF protocol was ACE activity and Ang-II levels.

Studies have shown that combination therapy with RF positively affects daytime blood pressure in hypertensive patients.^18^ On the other hand, conflicting results were reported in studies that found no difference.^19^ In addition, cardiovascular and metabolic benefits, such as the decrease in TG, LDL, fat mass, and CRP, have been reported in therapeutic fasting.^20^ Also, improvements in indicators of cardiovascular health could be seen 2–4 weeks after the start of IF in animal studies.^15^ In our study, compared to pre-IF, a significant decrease was observed in the mean systolic and diastolic blood pressures measured at 24 hours and night in post-IF hypertensive patients. In addition to the methodological differences in the studies, including controlled hypertensive patients in our study may have led to a more significant improvement in blood pressure levels since resistant cases were excluded. There was no statistically significant change in fasting blood glucose, HgA1C, creatinine, GFR, BUN, total cholesterol, LDL, HDL, and TG levels, while a significant decrease was observed in CRP levels after IF. Although, CRP levels were not evaluated as a predictive factor for improving blood pressure values in the regression analyses. Also, we observed no statistically significant change in BMI. In a meta-analysis study, it was reported that intermittent fasting had positive effects on BMI and fat ratio.^21^ On the other hand, considering the studies that diet times are mostly over 1 month, the reason why there was no significant change in BMI values in our study may be the duration. It is obvious that there is a need for new studies evaluating the effects of IF on BMI in the long term.

Patients with essential hypertension, especially those who are unmedicated, have an increase in sympathetic and a decrease in the parasympathetic activity of the autonomic nervous system.^22^ The RMSSD is the key time-domain metric used to assess the vagally mediated changes seen in HRV. It reflects the beat-to-beat variance in HR.^23^ RMSSD readings over 24 hours are highly associated with pNN50 and HF power.^24^ Limited studies investigated the effects of IF on HRV in patients with HT.^25 , 26^ In one study, HRV was assessed twice by ambulatory 24-hour Holter recordings at fasting during and after IF in 20 hypertension patients with sinus rhythm who varied in lifestyle factors. Considering the statistically significant variations in SDNN, SDANN, LF, and T power between the two groups, it was suggested that RF reduces the sympathetic nervous system’s activity.^25^ On the other hand, in another study that included 58 hypertensive patients, RF significantly increased HRV and reduced cardiac stress among patients controlled by an adherent to hypertensive medication.^25^ In our study, while a decrease was observed in Maximum Heart Rate after IF, Minimum Heart Rate and Night Average Heart Rate values decreased but did not change statistically. Compared to pre-IF, an increase in HF power and RMSSD levels and a decrease in LF/HF were observed in post-IF hypertensive patients. As an important result, RMSSD and HF power increases were assessed as predictive factors for decreasing systolic blood pressure after IF.

Ang-I is converted to Ang-II by the zinc metallopeptidase ACE.^27^ The effect of Ang-II on raising blood pressure and salt and water retention is well-known.^28^ Although animal studies reported that IF might have positive effects on RAS in the literature, no study assessed the effects of IF on RAS in hypertensive patients. In one animal study, Camelo et al.^29^ hypothesized that IF lowers blood pressure and improves lipid profile in mice due to a less activated local RAS in the left ventricle, regardless of the dietary plan.^29^ They found that the weight loss caused by the IF treatment resulted in regulation of the local RAS, with the benefit of left ventricular (LV) remodeling and blood pressure reduction.^29^ In another study with rats, it was investigated the effects of IF regimens on the plasma level of Ang-II, the expression of Ang-II receptors, and ACE2.^30^ The elderly animals were shown to have a higher heart hypertrophy index. The heart and aorta had higher AT1aR expression and less AT2R expression. Increasing the stated parameters and RAS balance, “every other day fasting” was more effective than “fasting one day each week.”^30^ In our study, serum Ang-I was found to be higher while Ang-II and ACE activity were found to be lower in the post-IF hypertensive patients than in the pre-IF hypertensive patients, and decreasing ACE activity and Ang-II levels were determined as predictive factors for decreasing systolic blood pressure after IF. Also, decreasing Ang-II level was a predictive factor for decreasing diastolic blood pressure after IF. Another point to be emphasized is that ACE activity is associated with BMI and thyroid functions.^31 , 32^ Therefore, euthyroid patients were included in our study, and no statistically significant change was observed in BMI and TSH levels before and after IF. To control these variables, BMI and TSH levels were added to the regression analysis in the model created for the change in blood pressure, and no significant relationship was found.

There are some limitations of our study. The number of participants was small, as only patients using dihydropyridine group calcium channel blockers were included in the study as antihypertensive treatment. Participants consisted only of patients with controlled hypertension and low cardiovascular risk. Future comprehensive studies are needed to determine whether IF applications are effective in patients with higher cardiovascular risk (such as diabetes, coronary artery disease, chronic kidney failure, and cerebrovascular disease) with higher RAS and ANS activity.

## Conclusions

In conclusion, the results of our study could be interpreted as:1. Parasympathetic system activation evaluated through HRV plays a role in the positive effects of IF on blood pressure. 2. Decreasing the Ang-II levels could be accepted as one of the reasons for improving hypertension over regulating sympathetic system activation over the RAS.
